# Supplementary material for: Deletion of wfs1 Impairs Oligodendrocyte Precursor Cells Dorsal Distribution and Myelination Through the wfs1-hmgcs1 Axis in Zebrafish
Source: Biology (Basel). 2026 Aug 16;15(16):1402. doi: 10.3390/biology15161402 (PMC13510151; doi:10.3390/biology15161402)
Supplement: Supplementary file 1 [file biology-15-01402-s001.zip › File S1-Transcriptome sequencing data and differentially expressed genes (DEGs) in wfs1-- zebrafish at 5 dpf/2.KEGG/wfs1KOvsWT/ALL/src/dre05132.html]

dre05132


- dre:562552

- Up regulated genes

562552(1.50682290794657)

- dre:100002946

- Down regulated genes

100002946(-1.40391573648291)

- dre:394198

- Up regulated genes

394198(1.20861445776772)
- dre:570238

- Up regulated genes

570238(2.66701017363204)

- dre:566185

- Up regulated genes

566185(6.72123660882883)

- dre:566185

- Up regulated genes

566185(6.72123660882883)

- dre:373874

- Up regulated genes

110437957(1.43280591760355)

- dre:386590

- Up regulated genes

386590\_1(9.5728659061572)

- Down regulated genes

386590(-2.05531811889352)

- dre:566185

- Up regulated genes

566185(6.72123660882883)

- dre:792699

- Down regulated genes

792699(-1.84389282152821)

- dre:792699

- Down regulated genes

792699(-1.84389282152821)

- dre:793158

- Up regulated genes

793158\_1(1.86160461223548)

- Down regulated genes

793158(-6.94943122774273)
- dre:556226

- Up regulated genes

556226\_1(6.36622858283894)

- dre:436718

- Up regulated genes

436718(1.34229427553561)

- Down regulated genes

436718\_1(-6.85571323128554)

- dre:393653

- Up regulated genes

393653\_1(6.66613004711253)

- dre:100002872

- Up regulated genes

100002872\_2(4.61563278220247)

- dre:57934

- Up regulated genes

57934\_1(9.91304974129429)

- Down regulated genes

57934(-1.25539807424297)

- dre:445305

- Down regulated genes

445305\_1(-1.98867996431405)

- dre:571356

- Up regulated genes

571356(1.35534919146634) 571356\_2(3.19114319937523)

- Down regulated genes

571356\_1(-1.36763102903653)

- dre:562372

- Up regulated genes

562372\_1(2.52467654758467)

- Down regulated genes

562372(-1.32469010475363)
- dre:558917

- Up regulated genes

558917\_1(1.2621896525325)
- dre:548603

- Up regulated genes

548603\_1(4.34052249142517)

- dre:321070

- Up regulated genes

101882911(3.50047007681359)

- dre:100004870

- Up regulated genes

100004870\_1(4.30187117398608)

- dre:393993

- Down regulated genes

novel.848(-2.69888694130701)

- dre:57934

- Up regulated genes

57934\_1(9.91304974129429)

- Down regulated genes

57934(-1.25539807424297)

- dre:564295

- Down regulated genes

564295\_2(-1.27307321576804)

- dre:373107

- Down regulated genes

373107\_1(-7.07514976036473)

- dre:561021

- Up regulated genes

561021(2.14284154145004)

- Down regulated genes

561021\_1(-4.68369619674599)

- dre:140621

- Down regulated genes

140621(-2.68196234303566) 140621\_1(-3.95107590777093)

- dre:436734

- Down regulated genes

436734\_1(-7.3292737184465)

- dre:562529

- Up regulated genes

562529\_1(8.14060403944833)
- dre:563023

- Up regulated genes

563023\_1(8.87487844876329)

- Down regulated genes

563023(-1.03169619245437)

- dre:57934

- Up regulated genes

57934\_1(9.91304974129429)

- Down regulated genes

57934(-1.25539807424297)

- dre:30429

- Up regulated genes

30429(3.98975094921512)

- Down regulated genes

30429\_2(-1.78714384880949)
- dre:677750

- Up regulated genes

677750(1.37913343503877)

- dre:403131

- Down regulated genes

403131(-4.42862381698358)

- dre:799806

- Down regulated genes

799806\_2(-2.00784502882026)

- dre:406297

- Up regulated genes

101882034(7.07443480885884)

- dre:431777

- Up regulated genes

431777(1.35055689210441)

- dre:336681

- Down regulated genes

406811\_1(-1.09337424046752)

- dre:436718

- Up regulated genes

436718(1.34229427553561)

- Down regulated genes

436718\_1(-6.85571323128554)

- dre:798985

- Up regulated genes

798985\_1(1.27260070507195)

- Down regulated genes

798985(-1.05520141640608)

- dre:431777

- Up regulated genes

431777(1.35055689210441)

- dre:336681

- Down regulated genes

406811\_1(-1.09337424046752)

Close
